# Polycytotoxic T cells mediate antimicrobial activity against intracellular *Mycobacterium tuberculosis*

**DOI:** 10.1128/iai.00297-24

**Published:** 2024-12-11

**Authors:** Marc Zumwinkel, Aaron Chirambo, Markus Zähnle, Max Bürger, Mark Grieshober, Vincent Romahn, Henry Mwandumba, Steffen Stenger

**Affiliations:** 1 Institute for Medical Microbiology and Hygiene, University Hospital Ulm 27197 , Ulm, Germany; 2 Malawi Liverpool Wellcome Trust Clinical Research Programme 560808 , Blantyre, Malawi; 3 Liverpool School of Tropical Medicine 9655 , Liverpool, United Kingdom; Stanford University School of Medicine, Stanford, California, USA

**Keywords:** CD8^+^ T lymphocytes, polycytotoxic, NKG2A, NKG2C, antimicrobial activity, tuberculosis

## Abstract

Protection against infections with intracellular bacteria requires the interaction of macrophages and T-lymphocytes, including CD8^+^ T cells. Recently, the expression of natural killer cell receptors NKG2A and NKG2C was introduced as markers of CD8^+^ T-cell subsets. The goal of this study was to functionally characterize human NKG2A and NKG2C-expressing T cells using the major pathogen *Mycobacterium tuberculosis* (*Mtb*) as a model organism. Sorted NKG2 populations were analyzed for their capacity to proliferate and degranulate and their intracellular expression of cytotoxic molecules. Cytokine release and the effect on bacterial growth were assessed after coculture of NKG2 populations with *Mtb*-infected macrophages. NKG2A^+^ T cells released higher levels of IFN-γ and IL-10, whereas NKG2C^+^ T cells released higher levels of IL-2, contained the greatest reservoir of intracellular granzyme B and showed a remarkable constitutive level of degranulation. Both subsets inhibited the intracellular growth of *Mtb* more efficiently than NKG2-negative CD8^+^ T cells. Antimicrobial activity of NKG2^+^ T cells was not associated with the release of cytokines or cytotoxic molecules. However, the frequency of polycytotoxic T cells (P-CTL), defined as CD8^+^ T cells co-expressing granzyme B, perforin, and granulysin, positively correlated with the ability of NKG2-expressing T cells to control *Mtb*-growth in macrophages. Our results highlight the potential of NKG2-expressing P-CTL to trigger the antibacterial activity of human macrophages. Targeting this population by preventive or therapeutic immune interventions could provide a novel strategy to combat severe infectious diseases such as tuberculosis.

## INTRODUCTION

To establish active infection, inhaled *Mycobacterium tuberculosis* (*Mtb*) must overcome the physical barriers of the upper respiratory tract and invade alveolar macrophages. Clearance of the pathogen from the lower airways requires a fine-tuned interaction of immune cells, most prominently T-lymphocytes and macrophages. There is definite evidence for the critical role of CD4^+^ T-lymphocytes in activating the antimicrobial activity of macrophages (1). The evidence for a contribution of CD8^+^ T cells in protection against *Mtb* is steadily increasing through conclusive animal studies and strong circumstantial evidence from human *ex vivo* and *in vitro* experiments (2–7). CD8^+^ T-lymphocytes promote the control of *Mtb* infection indirectly by the release of macrophage-activating cytokines or directly by the injection of cytotoxic molecules into infected macrophages (8). The granular molecules perforin, granzyme B, and granulysin interact to define an antimicrobial effector pathway leading to direct killing of intracellular bacilli (9). CD8^+^ T cells co-expressing all three effector molecules are called polycytotoxic T cells (P-CTL) (6). A subset of P-CTL expresses NK cell surface receptors (10, 11), which are categorized into Killer Cell Immunoglobulin-like Receptors (KIRs) and Natural Killer Group 2 (NKG2) Transmembrane Receptors. NKG2A and NKG2C form heterodimers with CD94 and bind to the non-classical major histocompatibility complex (MHC) molecule HLA-E (11). While the NKG2A receptor contains an immunoreceptor tyrosine-based inhibitory motif (ITIM) in its cytoplasmic domain, the NKG2C transmits an activating signal (11). Knowledge on the functional role of NKG2-expressing CD8^+^ T cells in infectious diseases is scarce (12). NKG2C^+^ T cells induce enhanced antimicrobial activity against the intracellular pathogens *Listeria monocytogenes*, *Mycobacterium leprae*, and the vaccine strain *Mycobacterium bovis Bacille Calmette Guerin* (10, 13). In addition, the low frequency of circulating NKG2C^+^ T cells was associated with active tuberculosis, further supporting a contribution of this subset in controlling infection (13). Based on these findings, we hypothesized that CD8^+^ NKG2-expressing T-lymphocytes provide functional support for macrophages in clearing the major human pathogen *Mtb*. To address this hypothesis, we cocultured sorted NKG2^+^CD8^+^ T cells with *Mtb*-infected human macrophages and characterized immune functions associated with protection against tuberculosis. The results define NKG2A and NKG2C T cells as two functionally distinct subsets. Both subsets are enriched for P-CTL (up to 50%) and the frequency of P-CTL correlated with the induction of antimycobacterial activity. We conclude that CD8^+^NKG2^+^ T-lymphocytes are a novel and attractive target for host-directed therapy in severe infections with intracellular bacteria, including tuberculosis.

## RESULTS

### Purification of functionally viable CD8^+^ T-lymphocytes expressing NKG2C or NKG2A

**Fig 1:**
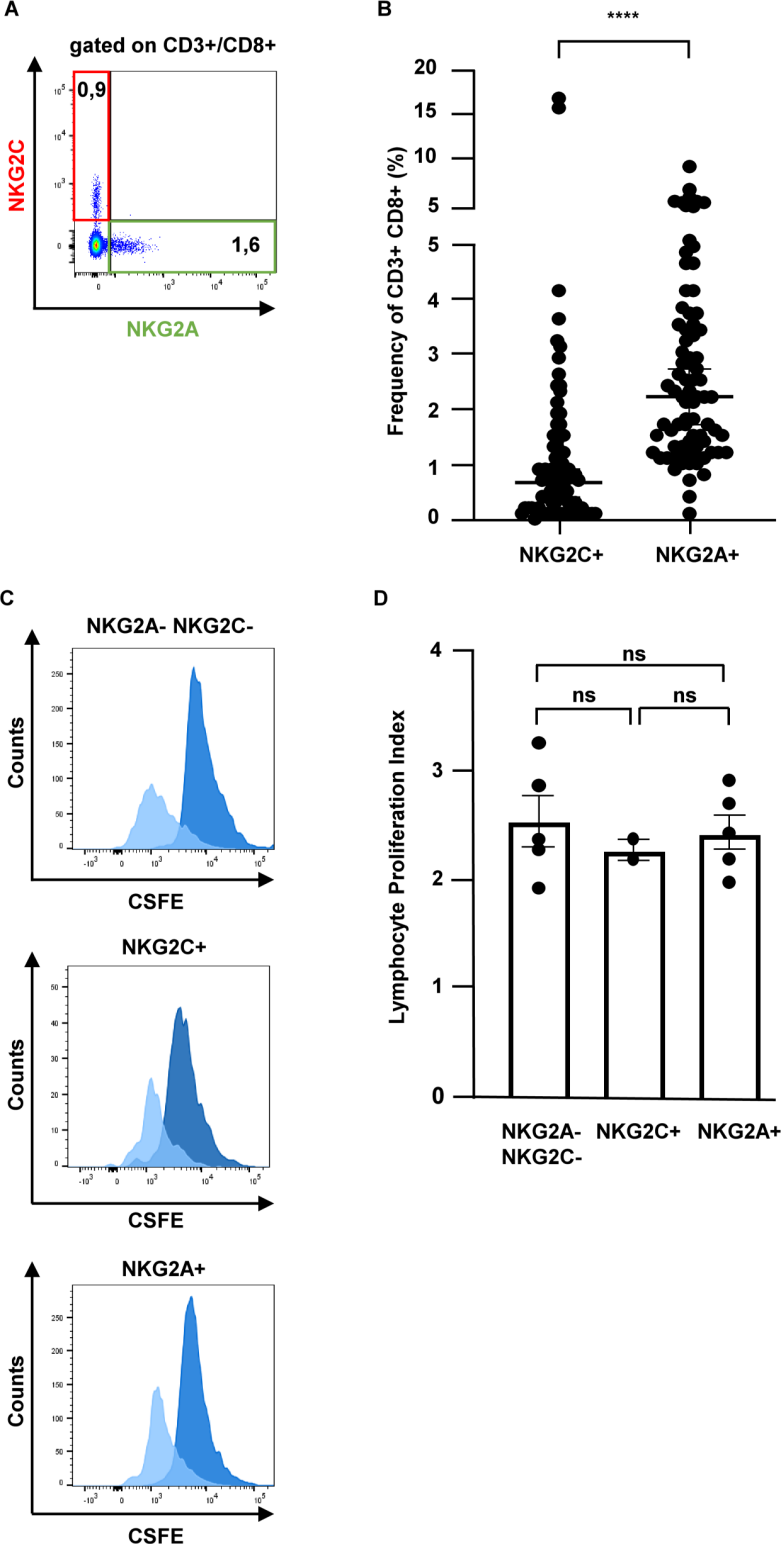
Sorting of functionally viable CD8^+^ T-lymphocytes expressing NKG2A or NKG2C. PBMCs were stained for CD3, CD8, NKG2A, NKG2C, and Live/Dead Fixable Viability Dye and analyzed by flow cytometry. (**A**) Representative dot plot from an individual donor (*n* = 80). (**B**) Frequency of NKG2C^+^ and NKG2A^+^ T cells within the CD3^+^CD8^+^ compartment for all 80 donors sorted (horizontal bar: median, Q_25_/Q_75_ are shown). Statistical analysis was performed using the Mann-Whitney test, two-tailed. (**C**) Sorted T cells were labeled with CFSE and stimulated for 6 days (lL-2/IL-7/IL-15; 10 ng/mL each). Fluorescence intensity was measured on Day 0 (dark blue) and Day 6 (light blue). Histograms from a representative donor are shown. (**D**) The lymphocyte proliferation index was calculated using the FlowJo proliferation algorithm (*n* = 5). The graph shows the mean and SEM. Statistical analysis was performed using the two-tailed Mann-Whitney test. PBMC, peripheral blood mononuclear cell.

The NKG2 transmembrane receptors NKG2C and NKG2A are implicated in shaping CD8^+^ T-cell-mediated immune responses against tumors and intracellular pathogens, including mycobacteria (10, 12, 13). We hypothesized that NKG2A or NKG2C are markers for human CD8^+^ T cell subsets with antimicrobial activity against intracellular *Mtb*. To test this hypothesis, we labeled peripheral blood mononuclear cell (PBMC) obtained from healthy donors with CD3, CD8, NKG2A, and NKG2C (Fig. 1A; Fig. S1). The frequency of CD3^+^CD8^+^ cells expressing NKG2A or NKG2C ranged between 0% and 16% in 80 donors tested (median: NKG2C: 0.6%, NKG2A: 2.2%) (Fig. 1B). Expression of NKG2A and NKG2C was mutually exclusive; hence, both receptors define a distinct subset of CD8^+^ T-lymphocytes. Cells were then sorted into CD3^+^CD8^+^NKG2A^+^, CD3^+^CD8^+^NKG2C^+^, and CD3^+^CD8^+^NKG2A^−^NKG2C^−^ cells by cell sorting reaching a purity of >98% (data not shown). The functional viability of sorted cells was high as indicated by a live/dead viability dye staining (data not shown) and the ability to proliferate in response to a cytokine cocktail containing IL-2, IL-7, and IL-15 (Fig. 1C). The lymphocyte proliferation index did not vary between the different T-cell populations (Fig. 1D). Despite a very low yield of T cells (10,000–80,000 cells/per group/per buffy coat), this robust protocol provided the basis for further functional studies of CD8^+^ T-cell subsets.

### Coculture of T-cell subsets and *Mtb*-infected macrophages: release of cytokines and cytotoxic molecules

**Fig 2:**
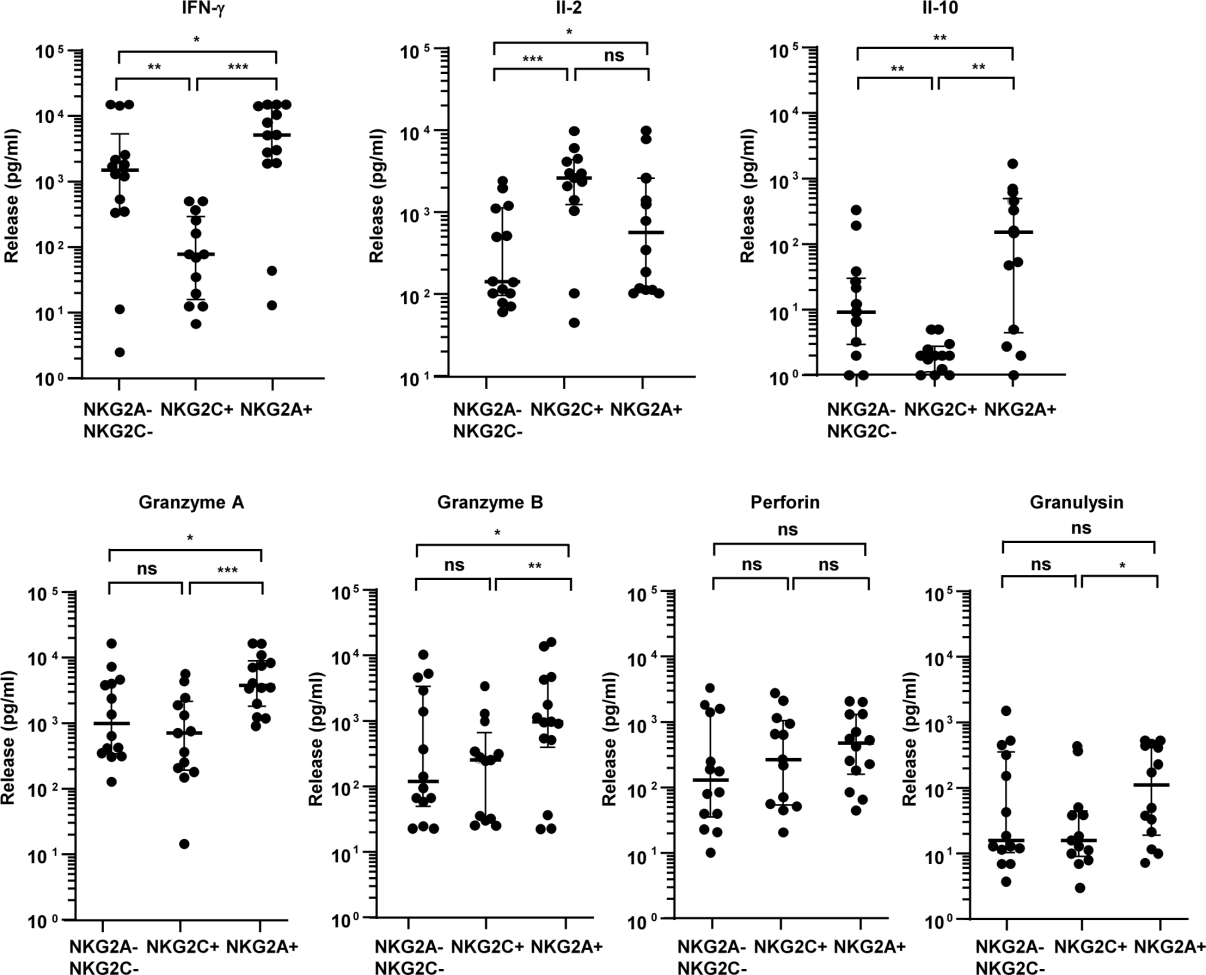
Coculture of purified T-cell populations and *Mtb*-infected macrophages: release of cytokines and cytotoxic molecules. Macrophages were infected with *Mtb*, coated with anti-CD3 antibodies and cocultured with sorted T cells for 24 h (ratio 1:1). The release of cytokines was measured using a cytometric bead assay. Each sample was measured in triplicates and the mean was calculated. The graph presents the release of cytokines from all 14 different donors tested. The median and Q_25_/Q_75_ are shown. Statistical analysis was performed using the Wilcoxon test, two-tailed. *Mtb*, *Mycobacterium tuberculosis*.

To define the functional characteristics of NKG2C- or NKG2A-expressing CD8^+^ T cells, we cocultured sorted cell populations with autologous macrophages infected with virulent *Mtb* H37Rv (ATCC 27294). Most of the donors included in our experiments had not previously been exposed to *Mtb* (as determined by *Mtb*-specific IFN-g-release assay) in agreement with the low incidence of latent tuberculosis in Germany. We therefore triggered an *Mtb*-independent T-cell activation using an anti-CD3 antibody in all experiments (10, 14). As correlates for protective immunity, we measured the cytokine release, the release of cytotoxic molecules, and the intracellular growth of mycobacteria after 24 h of incubation. The release of IL-2, IL-4, IL-6, IL-17A, IFN-γ, TNFα, soluble Fas, soluble FasL, granzyme A, granzyme B, perforin, and granulysin was determined using a bead-based immunoassay and analyzed by flow cytometry. The release of IL-6, IL-4, and soluble Fas was below the level of detection (9 pg/mL) in all groups (Table 1). NKG2A^+^ cells released significantly higher amounts of IFN-γ, IL-10, granzyme A, granzyme B, and granulysin than NKG2C^+^ and NKG2A^−^NKG2C^−^ cells (Table 1; Fig. 2). NKG2C^+^ cells released even less IFN-γ and IL-10 than NKG2A^−^NKG2C^−^ CD8^+^ T cells but were more potent producers of IL-2. These results demonstrate that NKG2A^+^ and NKG2C^+^ expressing CD8^+^ T-cell populations release different cytokines and cytotoxic molecules when cocultured with *Mtb*-infected macrophages.

**TABLE 1:** Coculture of sorted T-cell populations and *Mtb*-infected macrophages: release of effector molecules^*a*^

	NKG2A^−^NKG2C^−^	NKG2C^+^	NKG2A^+^
IFN-γ	1,500	78	5,200
IL-2	150	2,700	580
IL-10	<9	<9	150
IL-6	<9	<9	<9
IL-17A	22	21	50
TNFα	110	47	105
IL-4	<9	<9	<9
Soluble FasL	19	15	37
Soluble Fas	<9	<9	<9
Granzyme A	1,000	710	3,800
Granzyme B	120	250	970
Perforin	130	270	480
Granulysin	16	16	110

^*a*^ Supernatants were harvested after 24 h of coculture and the concentration of effector molecules was determined using a cytometric bead assay. Each sample was measured in triplicates and the mean was calculated. The table shows the median in pg/mL from 14 donors.

### Intracellular expression of granular molecules and degranulation of sorted T-cell populations

**Fig 3:**
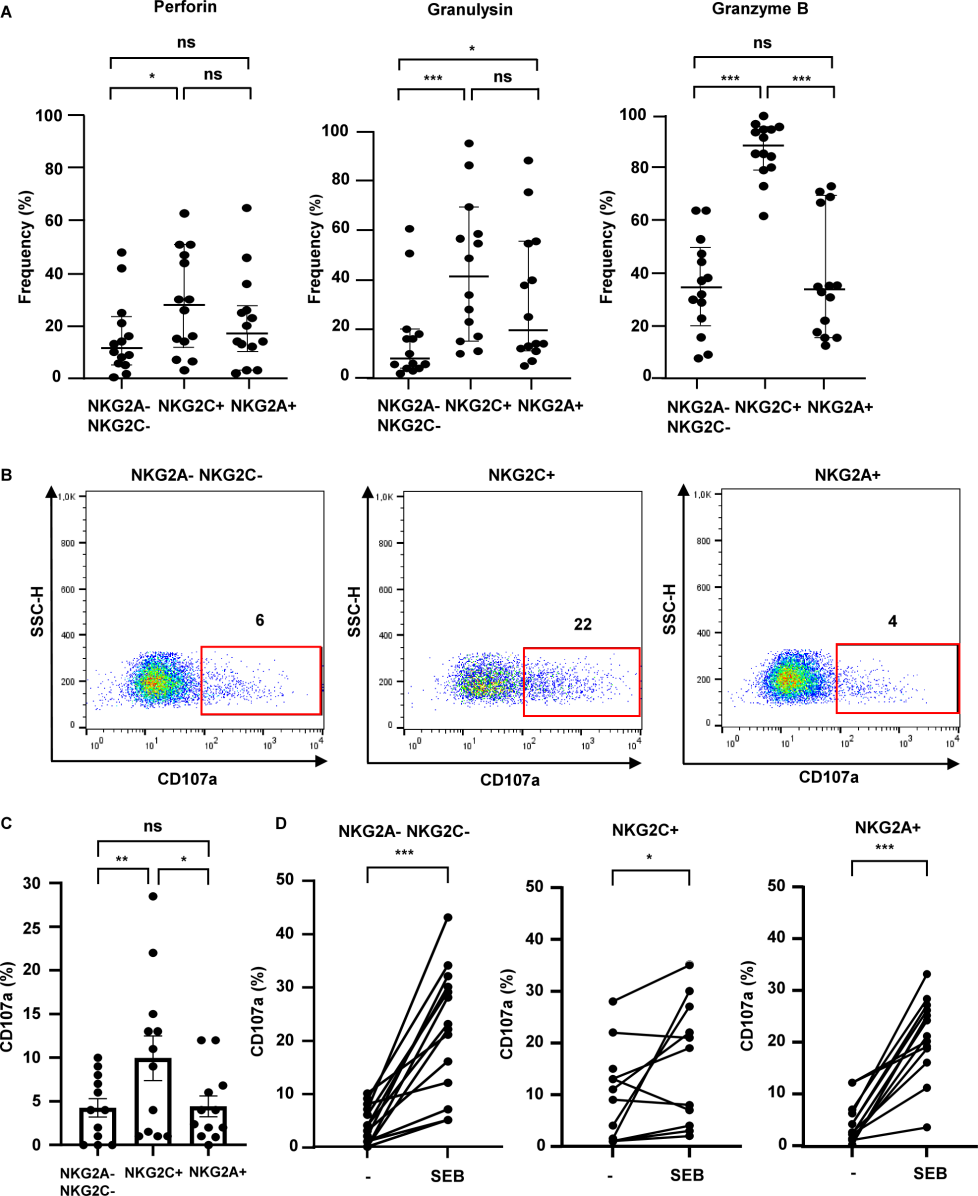
Intracellular expression of granular molecules and degranulation of purified T-cell populations. (**A**) The intracellular expression of granular molecules was determined by flow cytometry (*n* = 14). The median and Q_25_/Q_75_ are shown. Statistical comparison was performed using Wilcoxon test, two-tailed. (**B**) Sorted T-cell populations were labeled with anti-CD107a and cultured overnight in the presence of Monensin. Representative dot plots from all three subsets are shown. (**C**) Graph summarizes the frequency of CD107a-expressing un-treated cells from 14 independent donors. Mean and SEM are shown. Statistical comparison was performed using Wilcoxon test, two-tailed. (**D**) Comparison of CD107a-expression in un-treated- and SEB-treated cells (300 ng/mL) from 14 independent donors. Statistical analysis was performed using the Wilcoxon test, two-tailed.

Perforin, granulysin, and granzyme B are constitutively expressed in the granules of cytotoxic lymphocytes and act in concert to mediate an antimicrobial pathway (6, 9). Therefore, we measured the intracellular expression of perforin, granulysin, and granzyme B in the three subsets by eight-color flow cytometry. The frequency of all three molecules was significantly higher in the NKG2C^+^ T-lymphocytes as compared to the NKG2A^−^NKG2C^−^ T-lymphocytes (Fig. 3A). The median expression was also higher than in NKG2A^+^ lymphocytes, but only reached statistical significance for granzyme B. NKG2A^+^ T lymphocytes showed a higher frequency of intracellular granulysin expression when compared to NKG2A^−^NKG2C^−^ T cells.

To execute the cytolytic and antimicrobial function, granules must be translocated from the cytoplasm to the synaptic cleft between lymphocyte and target cells, for example, macrophages. Therefore, as the third functional criterion, we compared the CD107-expression in unstimulated and stimulated T-lymphocytes as a proxy for degranulation (Fig. 3B). The constitutive degranulation was moderate in NKG2A^−^NKG2C^−^ and NKG2A^+^ cells and significantly higher in NKG2C^+^ lymphocytes (Fig. 3C). SEB-stimulation significantly promoted degranulation in NKG2A^−^NKG2C^−^ and NKG2A^+^ subsets (Fig. 3D). The increase in the NKG2C^+^ subset—where we had observed pronounced constitutive degranulation—was less consistent. Co-incubation of CTL with *Mtb*-infected macrophages induced only low levels of CD107a translocation in NKG2A^−^NKG2C^−^ (from 1.5% to 6% on average) and NKG2A^+^ T cells (from 1% to 3% on average) and was therefore not informative for our study (data not shown).

### NKG2C^+^ and NKG2A^+^ T-lymphocytes inhibit the growth of intracellular *Mtb*

**Fig 4:**
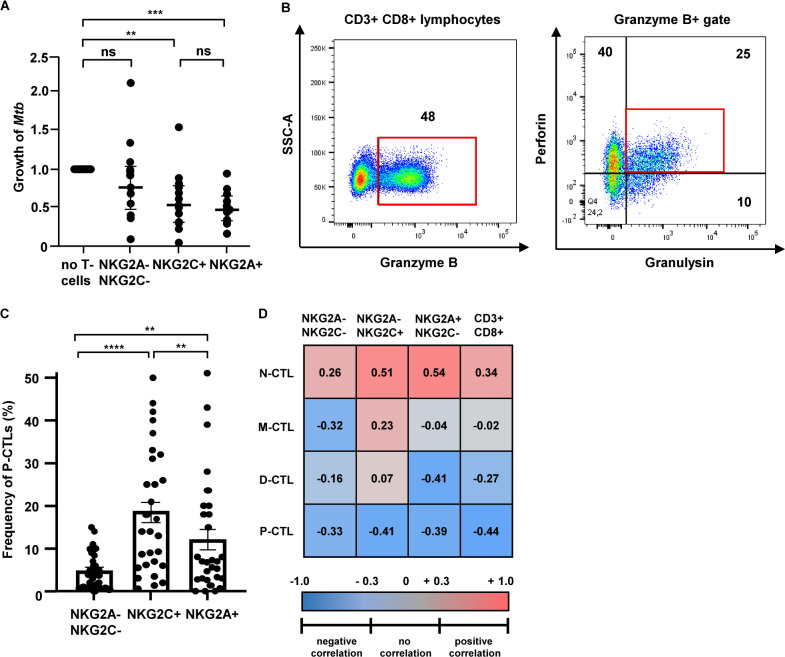
NKG2C^+^ and NKG2A^+^ T-lymphocytes inhibit the growth of intracellular *Mtb* and contain a higher frequency of P-CTLs. (**A**) Macrophages were infected with *Mtb*, coated with an anti-CD3 antibody, and cocultured with sorted T-cell subsets for 24 h. Lysates were plated in serial dilutions and the number of CFU was determined after 14 days of incubation. The number of CFU was determined and related to the number of CFU in the culture without T cells (defined as “1.0”). The graph shows the individual values of 13 independent donors (horizontal line: median). Statistical analysis was performed using the Wilcoxon test, two-tailed. (**B**) PBMCs from 30 donors were stained for CD3, CD8, NKG2A, NKG2C, granzyme B, perforin, granulysin, and Live/Dead and analyzed by flow cytometry. The Boolean gating function in FlowJo was used to determine combinatorial expression of granzyme B, perforin, and granulysin within all subsets. The dot plot illustrates the identification of P-CTLs using a subsequent gating strategy. The full gating strategy is depicted in S3. (**C**) The figure shows the frequency of P-CTL in all 30 donors investigated. Statistical comparison was performed with the Wilcoxon test, two-tailed. (**D**) Heatmap presenting the correlation between CTL-subsets and *n*-fold *Mtb* growth using Spearman’s rank correlation test (*n* = 12). *R*-values are depicted in the squares. CFU, colony-forming units; *Mtb*, *Mycobacterium tuberculosis*, P-CTL, polycytotoxic T-lymphocytes; PBMC, peripheral blood mononuclear cell.

After analysis of individual immunological parameters, we next determined the net effect of these functional modifications on the intracellular growth of *Mtb*. To investigate whether sorted NKG2C^+^ or NKG2A^+^ T cells activate human macrophages to restrict the growth of virulent *Mtb*, we cocultured infected macrophages with autologous T-cell subsets at a ratio of 1:1 and determined the number of viable bacilli after 24 h of incubation by plating cell lysates. NKG2A^+^ and NKG2C^+^ T cells significantly inhibited the growth of *Mtb* as compared to control cultures in the absence of T cells or NKG2A^−^NKG2C^−^ lymphocytes (Fig. 4A). NKG2A^+^ and NKG2C^+^ T cells were equally efficient reaching medians of 51% (NKG2C^+^) and 49% (NKG2A^+^), respectively.

### The frequency of polycytotoxic T cells correlates with the antimicrobial activity of CD3^+^CD8^+^ T-lymphocytes

We next attempted to define the mechanism(s) by which NKG2C^+^ and NKG2A^+^CD8^+^ T-lymphocytes limit *Mtb*-growth. The functional neutralization of multiple mediators by genetic manipulation was precluded by the low number of effector cells. To overcome this limitation, we performed statistical correlation between *Mtb*-growth and our previous immunological findings within the same cocultures of 13 donors. There was no significant correlation between *Mtb*-growth and the release of a single cytotoxic molecule or a cytokine (Fig. S2).

Cytotoxic T-cell-mediated antimicrobial activity requires the combined action of perforin, granulysin, and granzyme B. CD8^+^ T cells co-expressing all three molecules are named P-CTL (Fig. 4B) (6). Since we found no striking association between a single granular molecule and growth of intracellular *Mtb* in NKG2C^+^ or NKG2A^+^ T-lymphocytes, we determined the frequency of P-CTL as a possible predictive marker for triggering macrophage activation in sorted NKG2C^+^ or NKG2A^+^ cells from 30 healthy donors using a Boolean gating strategy (Fig. S3). The frequency of P-CTL was significantly higher in the NKG2C^+^ subset (19%) as compared to the NKG2A^+^ (12%) and NKG2A^−^NKG2C^−^ subset (5%) confirming our previous findings (Fig. 4C) (10). In 12 of these 30 donors, the cellular yield was sufficient to perform the complete set of experiments including *Mtb*-growth assays. In this cohort, we determined the frequency of CD8^+^ T-lymphocytes expressing no (N-CTL), one (M-CTL), two (D-CTL), or all three (P-CTL) granular proteins (Tables 2 and 3; Fig. S4). As expected, the frequency of N-CTL was highest in the NKG2A^−^NKG2C^−^ population. The percentage of D-CTL and P-CTL was highest in the NKG2C^+^ subset followed by the NKG2A^+^ T-lymphocytes confirming that the expression of NKG2 receptors is associated with the presence of cytotoxic molecules. In the CD3^−^CD8^+^NKG2^+^ population (e.g., a subset of natural killer cells), most cells expressed at least two cytotoxic molecules (Table 4). In 13 donors investigated, the frequency of D-CTL was 30% in both NKG2A^+^ and NKG2C^+^ cells. The frequency of P-CTL was 49% in the NKG2C^+^ subset and 40% in the NKG2A^+^ subset. These results demonstrate that the frequency of D-CTL and P-CTL is higher in the CD3^−^CD8^+^ than in the CD3^+^CD8^+^ population.

**TABLE 2:** Classification of CD3^+^CD8^+^ CTL-subsets^*a*^

	Perforin	Granzyme B	Granulysin
No cytotoxic molecules (N-CTL)	−	−	−
Mono-cytotoxic (M-CTL)	+	−	−
	−	+	−
	−	−	+
Di-cytotoxic (D-CTL)	+	+	−
	+	−	+
	−	+	+
Polycytotoxic (P-CTL)	+	+	+

^*a*^ CD3^+^CD8^+^ T cells were classified according to the number of cytotoxic molecules produced (perforin, granzyme B, and granulysin). Mono-cytotoxic T cells express one cytotoxic molecule, Di-cytotoxic T cells a combination of two cytotoxic molecules, and Polycytotoxic T cells all three molecules. +: present (expression >0.1% in staining), −: not present (detectable expression <0.1%). CTL: Cytotoxic T lymphocytes.

**TABLE 3:** Frequency of CTL-subsets in NKG2-sorted CD3^+^CD8^+^ T cells^*a*^

	N-CTL	M-CTL	D-CTL	P-CTL
NKG2A^−^NKG2C^−^	60	24	9	5
NKG2C^+^	6	34	33	21
NKG2A^+^	38	27	19	15

^*a*^ PBMCs were stained for CD3, CD8, NKG2A, NKG2C, granzyme B, perforin, and granulysin. The frequency of CTL-subsets was determined by flow cytometry. Numbers depict the median percentage of cells obtained from 13 different donors in %.

**TABLE 4:** Frequency of CTL-subsets in NKG2-sorted CD3^−^CD8^+^ T cells^*a*^

	N-CTL	M-CTL	D-CTL	P-CTL
NKG2C^+^	2,2	16	30	49
NKG2A^+^	11	16	30	40

^*a*^ PBMCs were stained for CD3, CD8, NKG2A, NKG2C, granzyme B, perforin, and granulysin. The frequency of CTL-subsets was determined by flow cytometry. Numbers depict the median percentage of cells obtained from 13 different donors in %.

**Fig 5:**
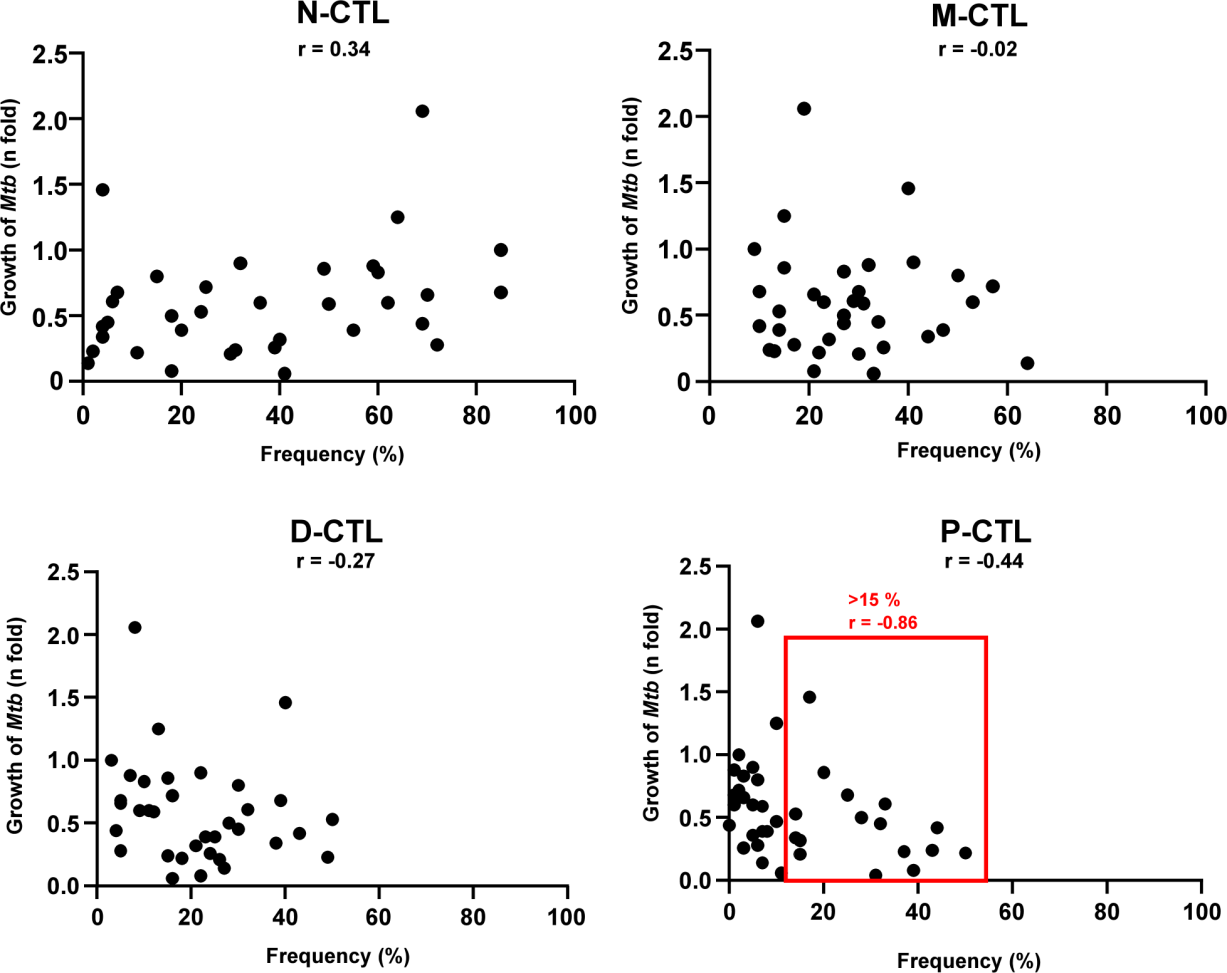
Correlation of the frequency of CTL-subsets and *Mtb*-growth. Correlation of mycobacterial growth (*n*-fold) to the frequency of P-CTL. Spearman’s rank correlation was used to measure the association between both variables and is indicated in the plot. The red box highlights donors with a frequency of P-CTL > 15%. The corresponding *r*-value is shown in red. P-CTL, polycytotoxic T-lymphocytes.

Since P-CTL are equipped with perforin, granulysin, and granzyme B, which define an antimicrobial pathway, we hypothesized that the frequency of this P-CTL correlates with the intracellular growth of *Mtb*. Strikingly, a high frequency of N-CTL positively correlated with the growth of *Mtb* in macrophages in all subsets (Fig. 4D). Coherent with our hypothesis, the frequency of P-CTL correlated with reduced intracellular *Mtb*-growth. Since this observation was independent of the expression of NKG2C or NKG2A, we considered the possibility that not the expression of NKG2 receptors, but the frequency of P-CTL is critical for controlling the growth of *Mtb*. To address this possibility, we re-analyzed our data and compared the frequency of CTL-subsets (N-CTL, M-CTL, D-CTL, and P-CTL) and the intracellular growth of *Mtb* independently of the expression of NKG2C or NKG2A (Fig. 5). We found a significant positive correlation between the frequency of N-CTL and the growth of *Mtb* (*r* = 0.34). In contrast, the frequency of P-CTL correlated with limited *Mtb* growth (*r* = −0.44). This finding was even more striking when the threshold for the frequency of P-CTL was arbitrarily set above 15%, where the correlation was nearly linear (*r* = −0.86). Taken together, these results indicate that independently of the expression of NKG2A or NKG2C, P-CTL contribute to the control of intracellular pathogens by human macrophages. Targeted amplification and activation of P-CTLs is therefore a promising strategy for optimizing the prevention and therapy of tuberculosis in humans.

## DISCUSSION

Our study provides novel insights into the distinct functional properties of CD8^+^ T-lymphocytes expressing the NK-cell receptors NKG2C or NKG2A in the context of *Mtb*-infection (summarized in Table 5). NKG2A^+^ cells release higher levels of IFN-γ, IL-10, and granzyme A, whereas the most prominent feature of NKG2C^+^ T cells is the capacity for constitutive degranulation. Both subsets inhibit the growth of intracellular *Mtb*, and this key function correlates with the frequency of cells co-expressing perforin, granulysin, and granzyme B (P-CTL). Targeting NKG2-expressing lymphocytes with an enriched frequency of P-CTL offers the intriguing possibility for host-directed immune interventions in tuberculosis.

**TABLE 5:** Summary of the phenotypic and functional properties of NKG2 subsets^*a*^

	NKG2A^−^NKG2C^−^	NKG2C^+^	NKG2A^+^
IFN-γ-release	++	+	+++
IL-10-release	−	−	+
IL-2-release	+	+++	+
Proliferation	++	++	++
Granzyme A-release	++	+	+++
Granzyme B-release	+	++	+
Degranulation, constitutive	−	++	−
Degranulation, stimulated	++	−	++
P-CTL	−	++	+
Inhibition of *Mtb*-growth	+	++	++

^*a*^ −: below detection limit; +: detected, <3-fold higher expression/release between the groups; ++: >3-fold higher expression/release between the groups; +++: >10-fold higher expression/release between the groups.

We performed our functional studies using sorted NKG2^+^ cells obtained from the peripheral blood of healthy donors. The cytokine release pattern, the expression of cytotoxic molecules, and the ability to trigger antimicrobial activity of macrophages are reminiscent of donor-unrestricted T cells (DURTs). These include HLA-E- and group 1 CD1-restricted T cells, mucosa-associated invariant T cells (MAITs), and γδ T-cell receptor-positive T cells (15). Functionally, HLA-E-restricted CD8^+^ T cells release IFN-γ, cytotoxic molecules and inhibit the intracellular growth of *Mtb* (16–20). Alternatively, NKG2^+^ T-lymphocytes could include T cells, which recognize lipid-antigens in the context of group 1 CD1 molecules (21). CD8^+^CD1-restricted T cells activated by lipoarabinomannan produce Th1-cytokines, are enriched in P-CTL, and activate macrophages to kill *Mtb* (6). Finally, MAIT cells and γδ T cells have very similar functional features as the NKG-expressing cells defined in this study (22–24). All four subsets of DURTs have been closely associated with protection or vaccine-induced prevention of tuberculosis infection in humans (25). Taken together, these studies suggest that non-classical, DURTs are involved in the prevention and protection from tuberculosis and NKG2C may be a surrogate marker to identify these antimicrobial T-cell subsets. This offers the intriguing possibility to strengthen immune responses by NKG2C ligation. In contrast to non-specific stimulation of NKG2C^+^ lymphocytes via IL-2 or IL-15, a more targeted approach could be to activate CD94, which forms a heterodimer with NKG2 molecules (11). Alternatively, the protective immune responses by NKG2C could be enhanced indirectly by neutralizing the function of NKG2A^+^ cells. NKG2A^+^ T cells contain an inhibitory ITIM motif and show lower antimicrobial activity against *M. leprae*, *L. monocytogenes*, and *M. bovis BCG* as compared to NKG2C^+^ T cells (10, 13). This is related to a lower frequency of P-CTL in NKG2A^+^ versus NKG2C^+^ T cells. The concept of an inhibitory function of NKG2A^+^ cells is supported by the higher frequency of this subset in the blood of patients with active tuberculosis as compared to healthy controls (13). In our experiments, NKG2A^+^ and NKG2C^+^ T cells were equally efficient in inhibiting mycobacterial growth. This somewhat conflicting finding could be explained by the different mycobacterial species and host cells used. While Shen et al. studied the growth of the highly attenuated vaccine strain *BCG* in a human macrophage-like cell line (THP-1), we focused on primary human macrophages infected with virulent *Mtb*.

This study confirms that NKG2C^+^ T cells are enriched for P-CTL. We show the first time, that NKG2C^+^ T cells support the growth restriction of virulent *Mtb* in human macrophages, extending earlier studies using attenuated mycobacteria (10, 13). The common concept evolving is, that P-CTL are an antimicrobial T-cell subset that is associated with protection against mycobacterial infections. The pore-forming perforin, the antimicrobial granulysin, and the proteolytic granzyme B define a pathway to support the control of infections with intracellular bacteria, fungi, and parasites (8, 9). Specific targeting of P-CTL as a novel strategy of host-directed therapy remains challenging because unique extracellular markers are still lacking. Even though NKG2C^+^ T cells are significantly enriched for P-CTL, the overall frequency is variable. Analysis of the antigen-specificity and the T-cell receptor repertoire of NKG2C^+^ P CTL are ongoing and will accelerate the development of this CD8^+^ T cell-subset as a target for the prevention and therapy of infections with mycobacteria.

## MATERIALS AND METHODS

### Antibodies and reagents

The following antibodies were used for flow cytometry: CD3-PerCP (clone SK7, BD Biosciences), CD8-BV605 (clone RPA-T8, BioLegend), NKG2A-FITC (clone REA 110, Miltenyi), NKG2A-PE (clone REA 110, Miltenyi), NKG2C-APC (clone REA 205, Miltenyi), granzyme B-Pacific Blue (clone GB11, BioLegend), GZMB-APC (clone GRB05, Invitrogen), perforin-PE-Cy7 (cloneB-D48, BioLegend), granulysin-Alexa488 (cloneRB1, BD Biosciences), granulysin-PE (cloneDH2, eBioscience), and CD107a-PE (BD Biosciences).

The following reagents and media were used for cell culture: Ficoll-PaquePLUS (GE Healthcare), Macrophage Serum-Free Media, AIM V (both from Gibco), rhu GM-CSF (Miltenyi), IL2 T6 (gift from Dr. Sallusto, Bellinzona, Switzerland), rec. IL-7 (R&D Systems), rec. IL-15 (R&D Systems), EDTA (Sigma-Aldrich), Paraformaldehyde (Sigma-Aldrich), Perm-Wash (BD Biosciences), CellTrace CFSE Cell proliferation Kit (Miltenyi), GolgiStop (BD Biosciences), Brefeldin A (Sigma-Aldrich), LEGENDplex Human CD8/NK Panel (13-plex) w/VbP V02 (BioLegend), Staphylococcus enterotoxin B (Sigma-Aldrich), 7H11 broth (BD Biosciences), Middlebrook OADC enrichment (BD Biosciences), Auramine-rhodamine (Merck), Tween 80 (Roth), and Glycerol (Sigma-Aldrich).

### Isolation of PBMCs and generation of macrophages: cell culture

Human PBMCs were isolated from buffy coats obtained from anonymous healthy individuals which donated whole blood for medical purposes using density gradient centrifugation (Institute of Transfusion Medicine, Ulm University Hospital, Germany), as previously described (26). All donors were tested negative for human immunodeficiency virus, Hepatitis B, Hepatitis C, and Cytomegalovirus by PCR. *Mtb*-specific IFN-γ release assays are not part of the routine screening of blood donors.

Plastic-adherent PBMCs enriched for monocytes were cultured in macrophage serum-free medium in the presence of granulocyte-macrophage colony-stimulating factor (GM-CSF, 10 ng/mL) for 6 days.

### T-cell proliferation: CFSE dilution assay

T cell proliferation was determined using the CFSE-dilution assay. Labeling was performed in AIM V with a final CSFE concentration of 0.5 µM. The cells were seeded in a 96-round bottom plate in AIM V supplemented IL-2, IL-7, and IL-15 (10 ng/mL). The cells were harvested on Day 0/6 and analyzed by flow cytometry (FACS Calibur, BD Biosciences, Franklin Lakes, USA). Quantitative data analysis was performed using the proliferation tool in the FlowJo Software package.

### Purification of NKG2C^+^ and NKG2A^+^ cells: cell sorting

CD8^+^ T cells were isolated from PBMC by magnetic beads separation according to the manufacturer's instructions (Miltenyi Biotec). After overnight culture, CD8^+^ T-lymphocytes were stained with Live/Dead fixable cell stain kit (Thermo Fisher Scientific, Waltham, USA), CD3-PerCP, CD8-BV605, NKG2A-FITC, and NKG2C-APC. CD8^+^ T-cell subsets were then sorted using BD FacsAria III. The purity was regularly >99%.

### Release of cytokines and cytotoxic molecules: LegendPlex

Cytokine-release in the supernatant by macrophage/T-cell cocultures was measured after 24 h of incubation using the LEGENDplex Human CD8/NK Panel (13-plex) w/VbP V02 (BioLegend, San Diego, USA) according to the manufacturer's instructions. All samples were measured in triplicates. The mean of all triplicates was used for further analysis. The sensitivity for the analytes ranged between 0.5 and 25 pg/mL.

### Degranulation of T-lymphocytes: CD107-staining

Sorted T-cell populations were seeded in a 96-well round-bottom plate in AIM V supplemented with cytokines for 5–6 days (IL-2, IL-7, and IL-15, 10 ng/mL each). Selected samples were stimulated with staphylococcal enterotoxin B (SEB, 300 ng/mL, 16 h). Anti-CD107a-PE (BD Biosciences) and GolgiStop were added to each well. After overnight incubation, CD107a expression was determined by flow cytometry.

### Detection of polycytotoxic T cells: intracellular flow cytometry

PBMCs were cultured at a concentration of 3 × 10^6^ cells/mL in six-well plates in AIM V supplemented with a cell culture supernatant enriched for IL-2 (IL-2T6). Sorted cells were cultured in 96-well round-bottom plates in a final volume of 200 µL AIM V supplemented with IL-2, IL-7, and Il-15 (10 ng/mL each). The cells were stained with the Live/Dead fixable cell stain kit, CD3-PerCP, CD8-BV605, NKG2A-PE, and NKG2C-APC. For intracellular staining, the cells were fixed with 4% paraformaldehyde, permeabilized (PermWash) and incubated with antibodies directed against granzyme B-PB, perforin-PECy7, and granulysin-Alexafluor 488). For each molecule, the positive population was determined by manual gating. The frequency of P-CTLs (live-single cells expressing CD3, CD8, perforin, granzyme B, and granulysin) was then calculated using the Boolean Gating function in FlowJo (Tree Star Inc., Ashland, USA).

### Mycobacteria

*Mtb* virulent strain H37Rv (ATCC 27294, Manassas, USA) was grown in suspension with constant rotation in roller bottles (Corning, NY, USA) containing 7H9 Middlebrook broth supplemented with 1% glycerol, 0.05% Tween 80, and 10% Middlebrook oleic acid, albumin, dextrose, and catalase enrichment. Aliquots from logarithmically growing cultures were sonicated to minimize clumping and frozen at −80°C in 7H9 broth with 20% glycerol. Representative vials were thawed and counted for viable colony-forming units (CFU) on the Middlebrook 7H11 plate (BD Biosciences). Staining of bacterial suspensions with fluorochromic substrates differentiating between live and dead bacteria (BacLight, Invitrogen, Carlsbad, USA) revealed a viability of the bacteria >90%.

### Measurement of intracellular *Mtb*-growth: CFU-assay

Before infection of macrophages, bacteria were thawed and sonicated in a water bath for 10 min to obtain a single-bacteria suspension. Macrophages were seeded in six-well cultures and infected with a multiplicity of infection (MOI) of 5. The rate of infection and cell morphology was determined using an auramine−rhodamine staining (Merck, Darmstadt, Germany). The rate of infection was donor-dependent and ranged between 20% and 30%. After 24 h, infected macrophages were resuspended in SFM. Macrophages were coated with an anti-CD3 antibody (20 ng/mL) for 15 min to support T-cell activation. 1 × 10^4^ infected macrophages were seeded in 96-well round-bottom plates and CD8^+^ T cells were added as indicated (effector:target ratio = 1:1). After 24 h of coculture, cells were lysed with 0.3% saponin. Cell lysates were resuspended vigorously and sonicated in a water bath for 10 min. Next, serial dilutions (1:10, 1:100, and 1:1,000) of the lysates were plated on 7H11 agar plates and incubated for 14 days before determining the number of CFU. Inclusion criteria for CFU counts were two dilutions with at least three colonies. Donors in which infected macrophages without T cells did not show intracellular growth were also excluded.

### Statistical analysis

For statistical evaluation a two-tailed, paired analysis was performed (Wilcoxon test). Due to the limited number of donors, we applied a non-parametric statistical analysis. *P* values < 0.05 were defined as significant and are indicated as follows: *: *P* ≤ 0.05, **: *P* ≤ 0.01, ***P ≤ 0.001, and ****: *P* ≤ 0.0001. All statistical analyses were performed using GraphPad Prism v8.2.1 (GraphPad Software).

## Data Availability

All raw data supporting the conclusions from this study are available upon request.

## SUPPLEMENTAL MATERIAL

Fig. S1 iai.00297-24-s0001.tif Sorting strategy.

Fig. S2 iai.00297-24-s0002.tif Correlation of single cytotoxic molecules to the growth of Mtb.

Fig. S3 iai.00297-24-s0003.tif Strategy to identify P-CTLs.

Fig. S4 iai.00297-24-s0004.tif Composition of CTL subsets.

Supplemental material iai.00297-24-s0005.docx Supplemental figure legends.

ASM does not own the copyrights to Supplemental Material that may be linked to, or accessed through, an article. The authors have granted ASM a non-exclusive, world-wide license to publish the Supplemental Material files. Please contact the corresponding author directly for reuse.
