# Supplementary figures and images for: Polycytotoxic T cells mediate antimicrobial activity against intracellular Mycobacterium tuberculosis
Source: Infect Immun. 2024 Dec 11;93(1):e00297-24. doi: 10.1128/iai.00297-24 (PMC11784352; doi:10.1128/iai.00297-24)

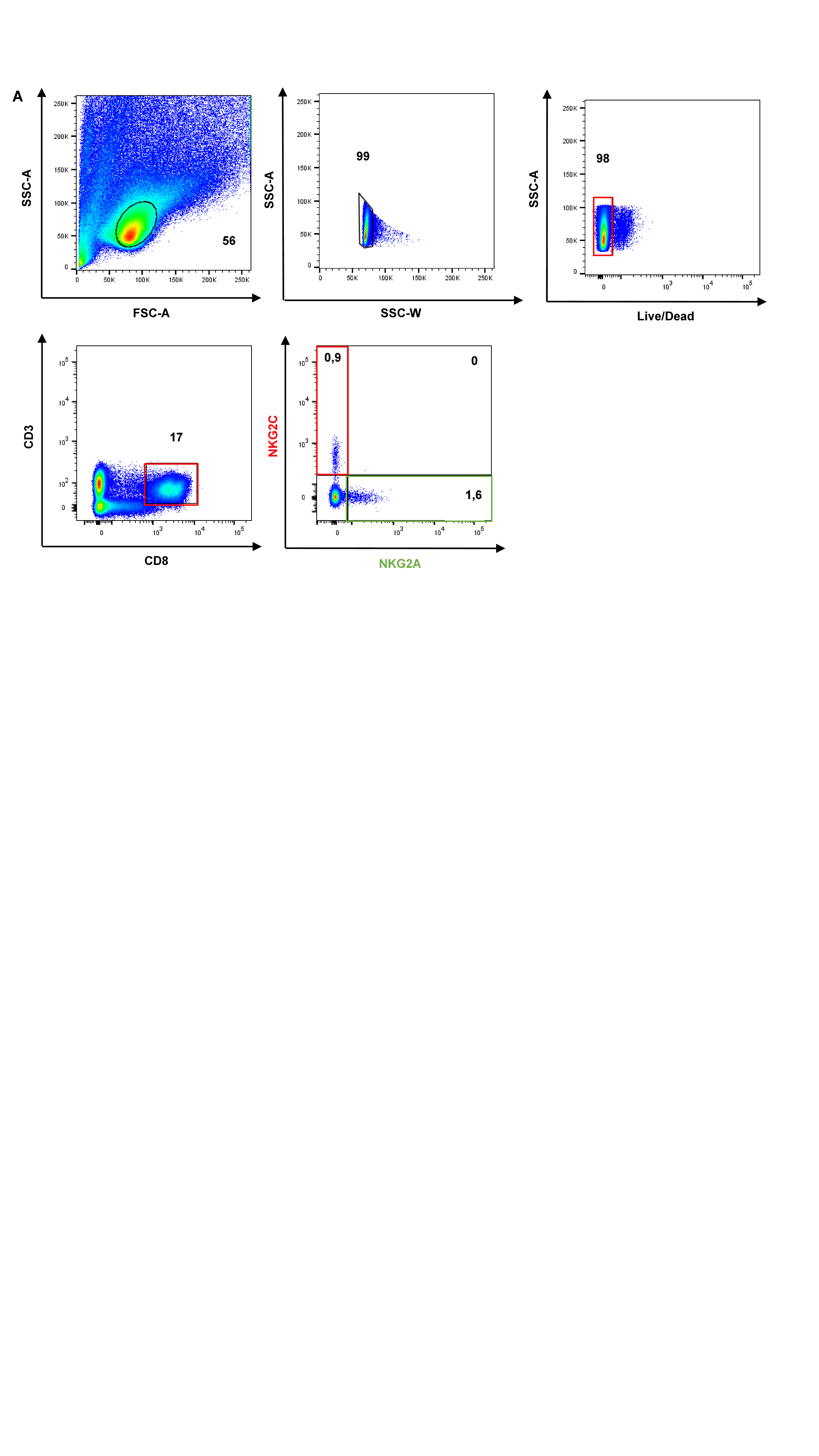

Supplement: Fig. S1 — Sorting strategy. [file iai.00297-24-s0001.tif]

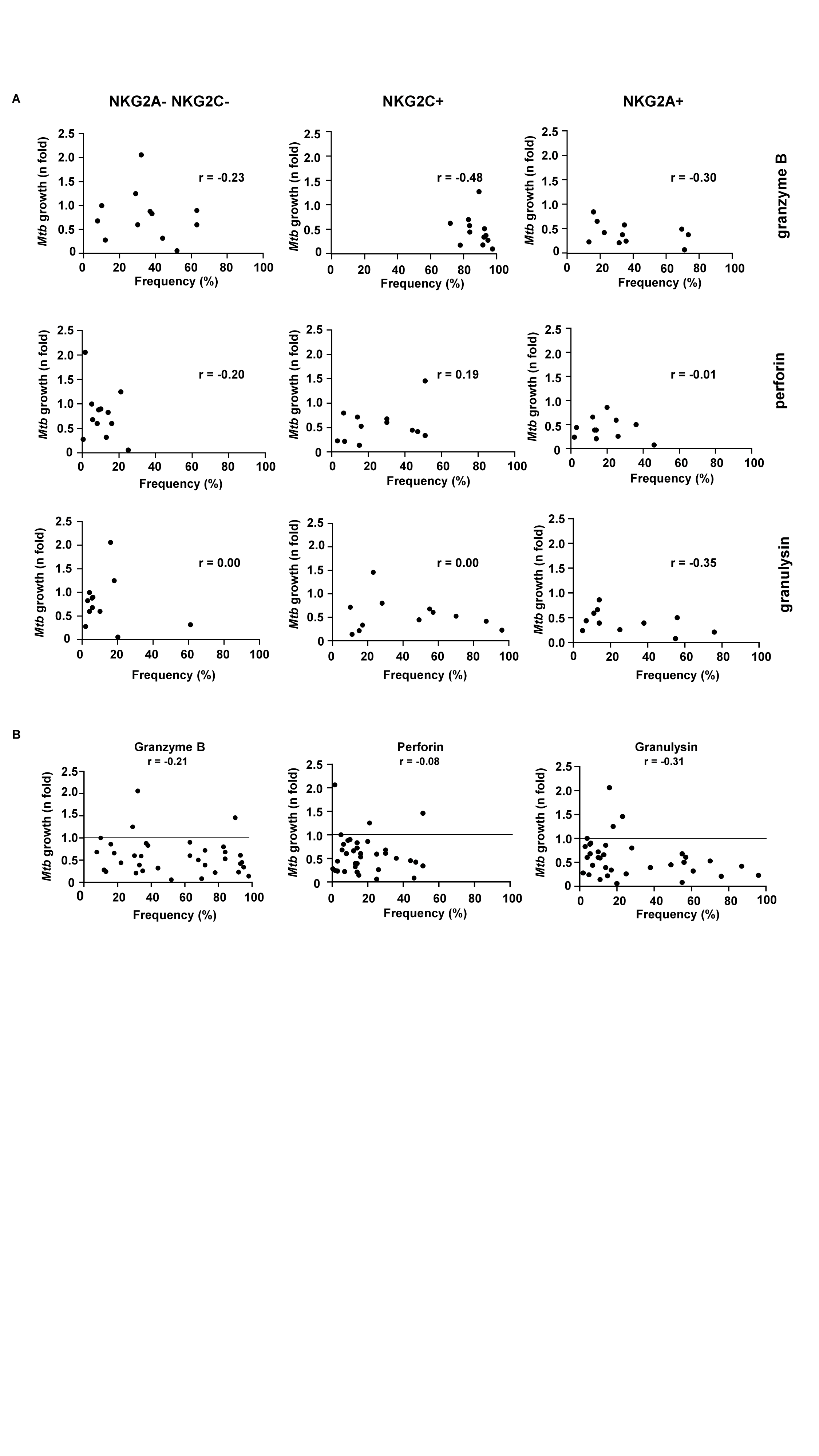

Supplement: Fig. S2 — Correlation of single cytotoxic molecules to the growth of Mtb. [file iai.00297-24-s0002.tif]

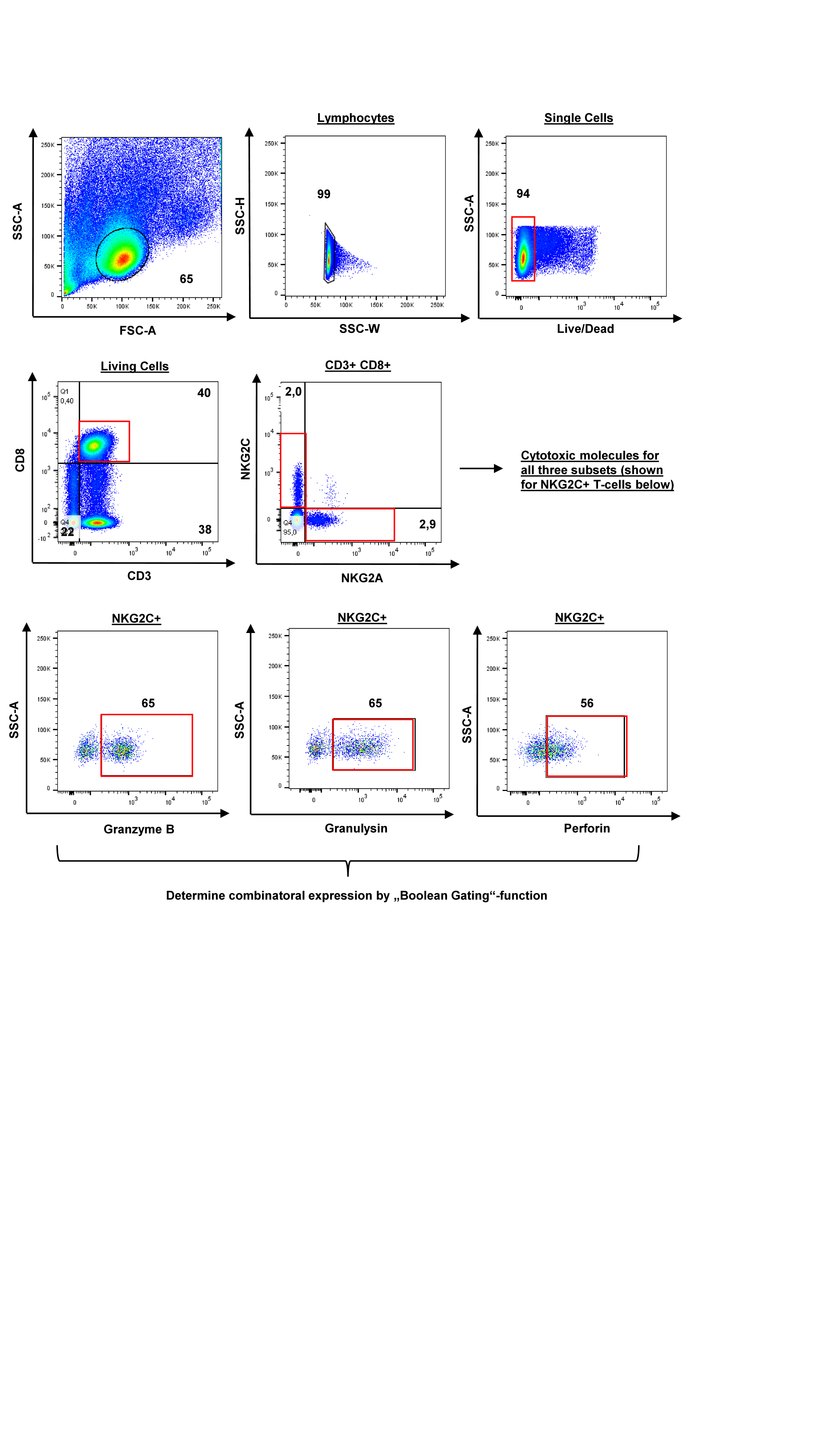

Supplement: Fig. S3 — Strategy to identify P-CTLs. [file iai.00297-24-s0003.tif]

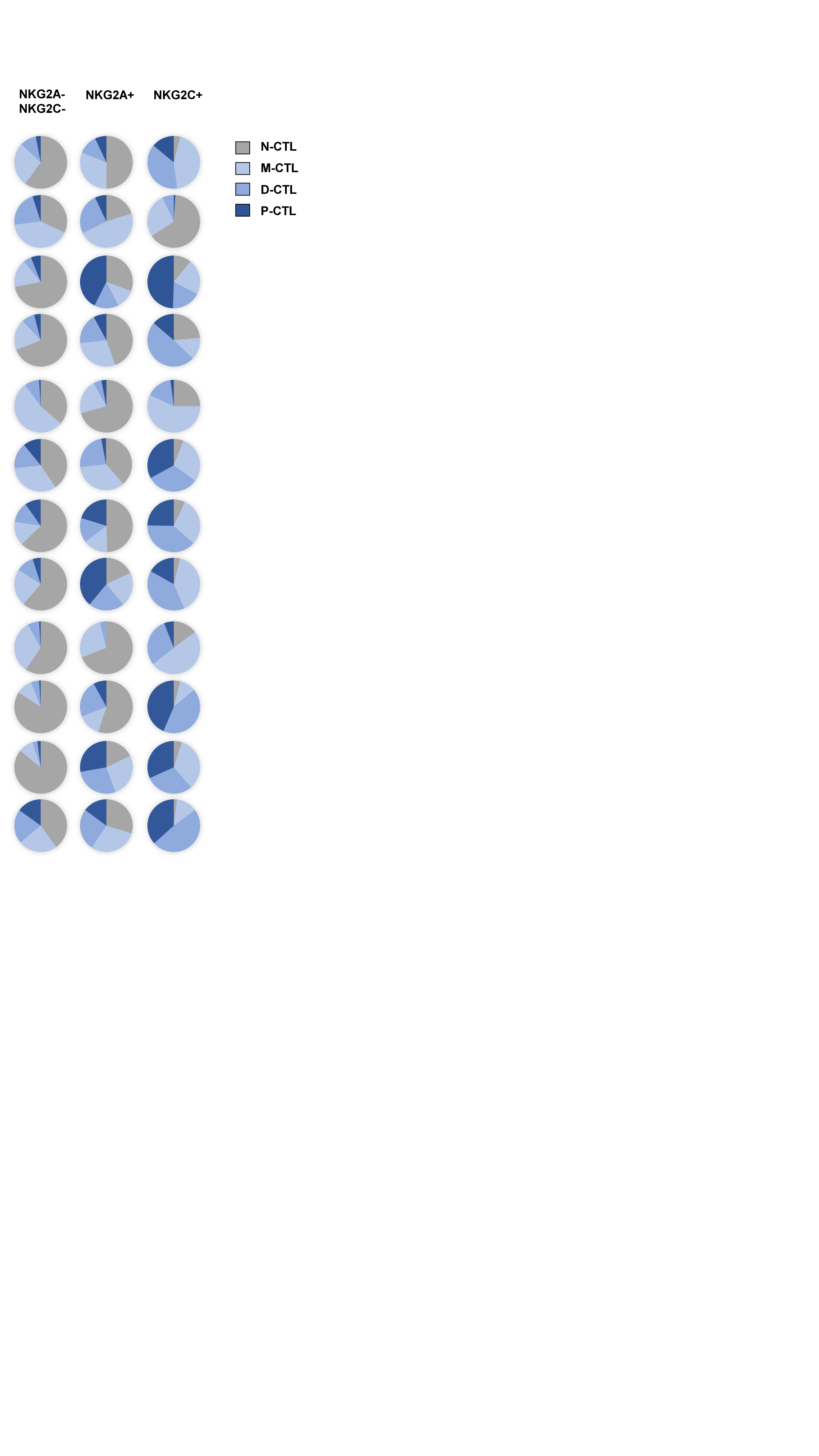

Supplement: Fig. S4 — Composition of CTL subsets. [file iai.00297-24-s0004.tif]
