# Supplementary material for: Polycytotoxic T cells mediate antimicrobial activity against intracellular Mycobacterium tuberculosis
Source: Infect Immun. 2024 Dec 11;93(1):e00297-24. doi: 10.1128/iai.00297-24 (PMC11784352; doi:10.1128/iai.00297-24)
Supplement: Supplemental material — Supplemental figure legends. [file iai.00297-24-s0005.docx]

**Supplementary Figure Legends**

**Supplementary Figure 1: Sorting strategy.** Gating strategy used to identify NKG2A+ and NKG2C+ T-cell subsets. Cells are gated from lymphocytes to singlets, living cells, CD3+ CD8+ T-cells and finally on the respective surface marker NKG2A+ or NKG2C+.

**Supplementary Figure 2: Correlation of the frequency of cells expressing granzyme B, perforin and granulysin and *Mtb*-growth**. The n-fold growth of *Mtb* was correlated with the intracellular expression of granular molecules in NKG2A-NKG2C-, NKG2C+ and NKG2A+ subsets **(A)** or compiled for all CTL-subsets **(B).** R-values were calculated using Spearman´s rank correlation test. *Mtb: Mycobacterium tuberculosis*, CTL: Cytotoxic T-lymphocytes*.*

**Supplementary Figure 3: Gating strategy used to identify P-CTL.** Cells are gated from lymphocytes to singlets, live cells, CD3+ CD8+ T cells and NKG2A+ NKG2C-, NKG2A- NKG2C- or NKG2A- NKG2C-. Within each subset production of the cytotoxic effector molecules granzyme B, perforin, and granulysin was assessed. Frequency of P-CTL were determined by Boolean Gating function. P-CTL: Polycytotoxic T-lymphocytes.

**Supplementary Figure 4: Frequency of mono-, di- and polycytotoxic T-cells in PBMC.** PBMCs from 12 donors were stained for CD3, CD8, granzyme B, perforin, granulysin and Live/Dead and analyzed by flow cytometry. The Boolean gating function in FlowJo was used to determine combinatorial expression. Pie Charts present the relative frequency of mono-, di- and polycytotoxic T-cells, as well as the percentage of T-cells that do not express cytotoxic molecules within CD3+/CD8+ T-cells. PBMC: Peripheral blood mononuclear cells.
